# Study of Isothermal, Kinetic, and Thermodynamic Parameters for Adsorption of Cadmium: An Overview of Linear and Nonlinear Approach and Error Analysis

**DOI:** 10.1155/2018/3463724

**Published:** 2018-07-03

**Authors:** Fozia Batool, Jamshed Akbar, Shahid Iqbal, Sobia Noreen, Syed Nasir Abbas Bukhari

**Affiliations:** ^1^Department of Chemistry, University of Sargodha, Sargodha 40100, Pakistan; ^2^Department of Pharmaceutical Chemistry, College of Pharmacy, Jouf University, Aljouf, Sakaka 2014, Saudi Arabia

## Abstract

Reports about presence and toxicity of Cd^2+^ in different chemical industrial effluents prompted the researchers to explore some economical, rapid, sensitive, and accurate methods for its determination and removal from aqueous systems. In continuation of series of investigations, adsorption of Cd^2+^ onto the stem of *Saccharum arundinaceum* is proposed in the present work. Optimization of parameters affecting sorption potential of Cd^2+^ including pH, contact time, temperature, sorbent dose, and concentration of sorbate was carried out to determine best suited conditions for maximum removal of sorbate. To understand the nature of sorption process, linear and nonlinear forms of five sorption isotherms including Freundlich and Langmuir models were employed. Feasibility and viability of sorption process were evaluated by calculating kinetics and thermodynamics of the process, while error analysis suggested best fitted sorption model on sorption data. Thermodynamic studies demonstrated exothermic nature of reaction, while kinetic studies suggested pseudo-second order of reaction.

## 1. Introduction

Environmental pollution should be taken into special consideration because it is a very serious matter affecting every type of organism at every level. The most adversely affected environmental resource is water [1]. As water is an essential element for the survival of living beings, it is very necessary to keep it pure and clean [2]. Quality of drinking water is of prime importance for mankind because waterborne diseases can decimate population of the whole area. These diseases arise due to toxic release of chemicals from industrial zones [3]. Particularly in industrial areas, these waterborne diseases are a great threat towards safety of water supplies. Other sources which may pollute water include domestic waste, pesticides run off from agricultural land, metal plating operations, and so on. Key contaminants present in water include heavy metals, chlorinated hydrocarbons, pathogens, detergents, pesticides, algal nutrients, trace organic compounds, dyes, and so on. These hazardous substances are of concern because of their ultimate effect on survival of human life [4]. Heavy metals like Cd, Zn, Ni, and Pb are present in relatively major amounts in industrial effluents and enter in rivers and oceans and ultimately pollute groundwater leading to adverse effects on aquatic life. Metals resist the process of biodegradability and hence remain in ecosystem, affecting food chain and human health [5]. In order to provide clean environment and healthy lifestyle to our coming generations, it is necessary to remove hazardous pollutants from the environment. In environmental restoration areas, conventional techniques are practiced to eradicate those pollutants from the environment which include chemical precipitation, evaporative method, electrolytic extraction, reverse osmosis, ion exchange, and electrochemical and membrane processes [6]. All these methods are costly and produce large amount of sludge which is difficult to dispose of. Use of biological materials for the removal of pollutants from aqueous media is considered superior to other methods in terms of cost effectiveness and simple design. It is a surface phenomenon, in which pollutants get accumulated on the surface of the adsorbent material. Binding nature is based on type of sorbent and sorbate, but mostly physisorption or chemisorption takes place [7]. Materials with ease in availability and low cost are preferred for the purpose. In this context, agrowastes are considered a significant material for adsorption. Binding capacity of these materials can be intensified by physical and chemical treatments and heat therapy [8].

To explore the appropriate adsorbent, it is necessary to establish equilibrium correlation of sorbent to predict behavior of sorbent under different experimental conditions. This equilibrium correlation is developed by using equilibrium isotherms. These isotherms express way of sorbent interaction with the surface of adsorbent, that is, whether it is monolayer or multilayer sorption [9]. Similarly, thermodynamic studies are of prime importance to predict whether the adsorption is spontaneous or not. Furthermore, it provides information about suitable temperature range for sorption and nature of sorbent and sorbate at equilibrium [10].

The aim of the present research was to explore *Saccharum arundinaceum* for adsorption of cadmium under different operating conditions including pH, contact time, initial concentration, and temperature. The application of linear and nonlinear forms of equilibrium isotherms was to determine appropriate isotherm for the purpose, and thermodynamic and kinetic studies were performed to check reaction nature of the adsorption phenomenon. Error analysis based on five different error functions was also performed.

## 2. Materials and Methods

### 2.1. Preparation of Adsorbent

On the basis of literature survey and indigenous availability of agrowaste materials, the stem of *Saccharum arundinaceum* (hardy sugar cane) was collected from different regions of Sargodha District, Pakistan. After collection, the sample was properly washed with deionized water to remove dust and surface impurities. The sorbent was initially dried in an open container at room temperature and later in an electric oven (Model, LEB-1-20) at 105°C for 24 h to remove all the moisture contents. The dried sorbent was ground, and appropriate particle size was separated by sieves and was stored for further analyses.

### 2.2. Chemicals

All the chemicals, reagents, and solvents used in the present work were of analytical reagent grade and purchased from Merck (Germany) or Sigma-Aldrich (Germany). Standard solutions were prepared, and successive dilutions were made with double-distilled water to make working solutions.

### 2.3. Pretreatment of Sorbents

*Saccharum arundinaceum* was pretreated with HCl (0.1 M) and NaOH (0.1 M) to evaluate the effects of acid and base treatments on pore size, that is, pore area, pore volume, and sorption capacity. For chemical treatment, the sorbent (20 g) was stirred for 4 h in 1 L solution of 0.1 M NaOH or HCl followed by filtration and extensive washing with distilled water to remove any traces of acid/base. After that, the treated sorbent material was dried at 110°C and stored in airtight zipper bags at −4°C before further use.

### 2.4. Characterization of Sorbent

To determine different physical and chemical parameters affecting adsorption, it is necessary to characterize the sorbent. Therefore, physical and chemical characterization was done by scanning electron microscopy (SEM) and Fourier transform infrared spectroscopy (FTIR).

#### 2.4.1. Scanning Electron Microscopy

Surface analysis was performed using scanning electron microscope JEOL model 2300. SEM provides information about surface area available for adsorption and morphology of sorbent [11]. Analysis of each sorbent was carried out in optimized conditions under argon atmosphere.

#### 2.4.2. Fourier Transform Infrared Spectroscopy

Functional groups present in structure of sorbent were determined by Fourier transform infrared spectrophotometer (Model Shimadzu AIM-8800). These functional groups are responsible for adsorption of sorbate on the surface of sorbent, and their detection helps in determining the nature of binding interactions between sorbate and sorbent surface [12]. Diffused reflectance infrared technique (DRIFT) was used for analysis taking KBr as a background reagent.

### 2.5. Equilibrium Isotherms

In order to study adsorption pathway and equilibrium relationship between sorbent and sorbate, it is necessary to design proper adsorption isotherms. Isotherms predict the appropriate parameters and behavior of sorbent towards different sorption systems [13]. In this context, linear and nonlinear models are utilized using Microsoft Excel®2007 (equilibrium isotherms applied on the present work are given in Table 1s of supplementary data).

### 2.6. Error Functions

In order to determine best fitting of linear or nonlinear models onto adsorption data, it is necessary to calculate the error function [14]. These error functions include sum square error, hybrid functional error, average relative error, sum of absolute error, nonlinear chi-square, and so on (calculated error functions and their equations are present in Table 2s of supplementary data).

### 2.7. Thermodynamic Investigations

Thermodynamic investigations are another important parameter of adsorption studies. For thermodynamic studies, the adsorption experiment was carried out at different temperature conditions and calculated parameters included enthalpy (Δ*H*), entropy (Δ*S*), and Gibbs free energy (Δ*G*).

For this purpose, (1)–(3) were applied

(1) $$\begin{array}{c} \Delta G^{{}^{\circ}}=\Delta H^{{}^{\circ}}-T\Delta S^{{}^{\circ}}, \end{array}$$

(2) $$\begin{array}{c} \ln\;K_{C}=-\frac{\Delta H}{RT}+\frac{\Delta S}{R}, \end{array}$$

(3) $$\begin{array}{c} \Delta G=-RT\text{⁡}\ln\text{⁡}K_{C}, \end{array}$$

where *R* is the natural gas constant and *K*_C_ is the constant at equilibrium and is calculated as

(4) $$\begin{array}{c} K_{C}=\frac{C_{e}}{1-C_{e}}. \end{array}$$

where *C*_e_ is the concentration of sorbent at equilibrium condition.

### 2.8. Adsorption Kinetics

In batch adsorption process, kinetic studies provide information about optimum conditions, mechanism of sorption, and possible rate controlling step. For this purpose, linear and nonlinear form of pseudo-first- and pseudo-second-order kinetics is applied on adsorption data [15]. In order to check the effect of contact time (10–70 min) on adsorption, initial concentration of 100 mg/L for cadmium was prepared and 100 ml of this sample was used for study. Sorbent (0.5 g) was added in this cadmium solution and applied for shaking at 150 rpm speed. After fixed interval of time, the sample was removed from flask and analyzed for cadmium concentration by atomic absorption spectrophotometer. The amount of cadmium adsorbed at different time intervals was calculated by employing the following formula:

(5) $$\begin{array}{c} Q_{t}=\frac{\left(Q_{o}-Q_{e}\right)}{W_{\text{sorbent}}}\text{⁡}\times V, \end{array}$$

where *Q*_*t*_ is the amount of cadmium adsorbed at any time *t*, *Q*_o_ and *Q*_e_ are initial and equilibrium concentrations, respectively. The volume of cadmium solution taken is represented by *V*(*L*), and *W*_sorbent_ is the amount of sorbent in g.

#### 2.8.1. Pseudo-First-Order Kinetics

In order to calculate pseudo-first-order kinetics for adsorption system, following equations were used:

(6) $$\begin{array}{c} \ln\left(Q_{e}-Q_{t}\right)=\ln\left(Q_{e}\right)-k_{1}t\;\;\mathrm{linear}\;\;\mathrm{form,}\\ Q_{t}=Q_{e}\left(1-e^{-k_{1}t}\right)\;\;\mathrm{nonlinear form,} \end{array}$$

where *Q*_*t*_ is the amount adsorbed at time *t*, *Q*_e_ is the equilibrium amount, *t* is time in minutes, and *k*_1_ is the rate constant.

#### 2.8.2. Pseudo-Second-Order Kinetics

For pseudo-second-order kinetics, linear and nonlinear forms were applied as follows:

(7) $$\begin{array}{c} \frac{t}{Q_{t}}=\frac{1}{k_{2}Q_{e}^{2}}+\left(\frac{1}{Q_{e}}\right)t\;\;\mathrm{linear}\;\;\mathrm{form,}\\ Q_{t}=\frac{k_{2}Q_{e}^{2}t}{1+k_{2}Q_{e}t}\;\;\mathrm{nonlinear}\;\;\mathrm{form.} \end{array}$$

## 3. Results and Discussion

### 3.1. Effect of Pretreatment

**Figure 1:**
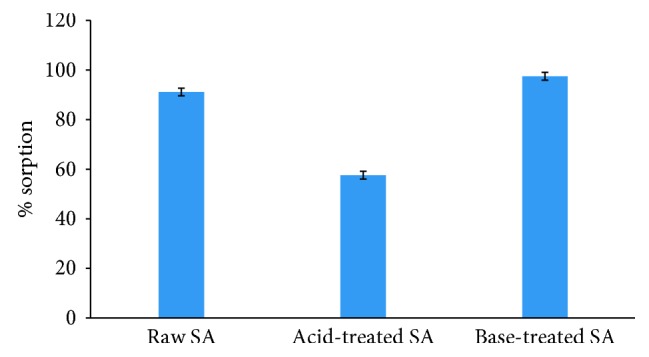
Effect of pretreatment on adsorption capacity of *Saccharum arundinaceum* (0.1 M HCl and NaOH treated sorbent, 60 minutes time, and 60 ppm initial concentration).

Pretreatment has promising effect on adsorption potential of *Saccharum arundinaceum*. Results reveal that base-treated (97.5%) sorbent shows good efficiency for cadmium sorption as compared to raw (91.15%) and acid-treated (57.6) sorbent as shown in Figure 1. Adsorption capacity depends upon functional groups present on the surface of sorbent and its microporous structure [16]. Increase in sorption capacity by base treatment can be attributed to hydroxyl groups created on the surface of adsorbent by base treatment or modification of cell wall components by base [17]. Decrease in adsorption after acid treatments was found as the binding sites available on the surface of biosorbent got destructed due to acid [18]. So, base-treated *Saccharum arundinaceum* was used for adsorption analysis.

### 3.2. Characterization of Sorbents

*Saccharum arundinaceum* was characterized in terms of surface morphology and functional group analysis by scanning electron microscopy and Fourier transform infrared spectroscopy.

#### 3.2.1. Scanning Electron Microscopy

**Figure 2:**
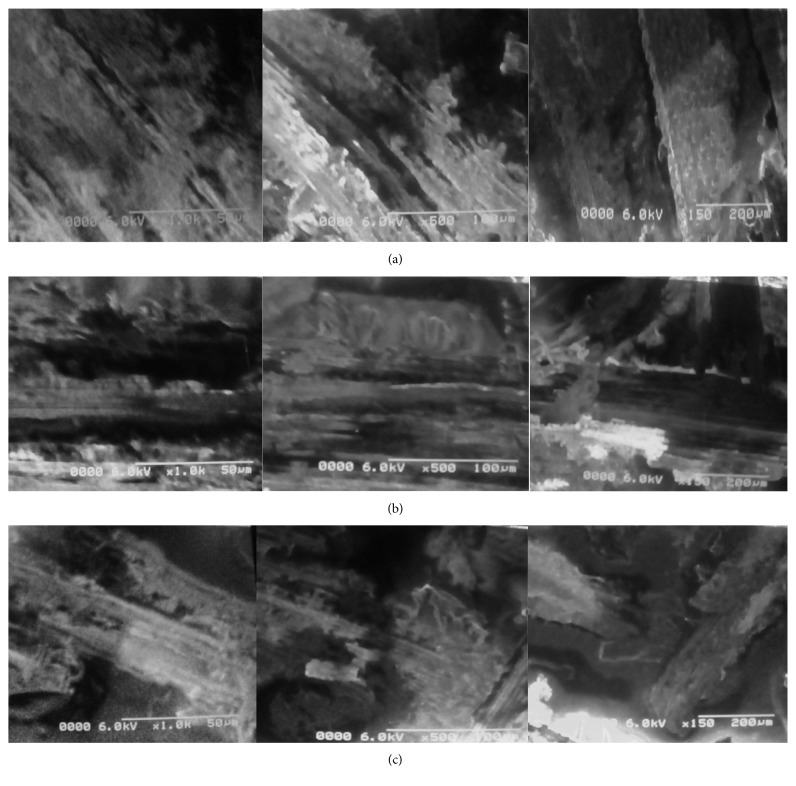
(a) SEM image of raw *Saccharum arundinaceum* at three different resolutions. (b) SEM image of base-treated *Saccharum arundinaceum* at three different resolutions. (c) SEM image of acid-treated *Saccharum arundinaceum* at three different resolutions.

Three native and two treated sorbents (acid- and base-treated *Saccharum arundinaceum*) were analyzed through scanning electron microscope to study surface morphology. Large pore size available on the surfaces of native and base-treated sorbent was responsible for enhanced adsorption on the surface of these agrowaste materials. Results for SEM analysis are given in Figure 2. Hollow cavities appear in the structure of raw adsorbent, which were responsible for binding of sorbate onto the surface of sorbent. Acid treatment decreases these cavities by deforming surface of the sorbent, so adsorption decreases after acid treatment because surface becomes smooth and thin adsorption layer is formed on the sorbent surface. Raw and base-treated sorbent surface is found rough and cylindrical, which make possible multilayer and thick adsorption on the sorbent surface as compared to smooth surface. Results obtained in the SEM micrograph are in good agreement with reported data [19].

#### 3.2.2. Fourier Transform Infrared Spectroscopy

Fourier transform infrared spectrometer provides information about functional groups present on the surface of sorbent and makes possible attachment of sorbate [20]. FTIR spectra of sorbent obtained in the range of 4000–450 cm^−1^ wavenumber and major functional groups present in adsorbent are listed in Table 1. A broadband appears in the range of 3000–3700 cm^−1^ which was due to –OH stretching vibration of hydroxyl functional groups including hydrogen bonding because broadband of –OH group in this range is indication of hydrogen bonding present in the compound. This peak appears in raw and base-treated sorbent but disappears in case of acid treated due to reaction of –OH group with acid hydrogen. Stretching band of –CH appears in 2900–3000 cm^−1^ wavenumber range for all sorbents. The peak at 1750 cm^−1^ appears due to C=O and 1200 cm^−1^ due to C-O functional group. In some cases, –CN also appears at 1049 cm^−1^ value. Vibration due to secondary amide appears at 1645 cm^−1^ [21]. For adsorption purpose, significant role is played by –OH group and heteroatoms to attach sorbate on the surface.

### 3.3. Adsorption Study

Adsorption study was performed by the batch adsorption method by varying different parameters including pH, contact time, and initial concentration of sorbate to find best suited conditions for the removal of cadmium from aqueous media.

#### 3.3.1. Effect of Contact Time

Contact time was varied from 10 to 100 minutes under neutral conditions with constant amount of sorbent (1 g), initial concentration (60 ppm), and shaking speed (150 rpm), and results obtained are shown in Figure 1s (Supplementary material).

Maximum adsorption was achieved at 60 minutes time interval and no significant increase found by further increase in time. Initially, excess of vacant places are available on the surface of sorbent, and uptake of metal ions was more, so there was continuous increase in adsorption capacity by increasing time slot from zero to 60 minutes. But, further increase could not cause sufficient change in adsorption of metals as vacant spaces are already filled, and equilibrium is achieved [22].

#### 3.3.2. Effect of pH

Initial pH of the adsorption system has significant role in adsorption of sorbate, as it affects the surface morphology of the sorbent and binding nature of sorbate. The range of pH selected was 2–10 with 1 g sorbent and 60 ppm initial concentration of sorbate. The result given in Figure 2s (Supplementary material) reveals the fact that adsorption capacity is quite low under acidic conditions. When pH is increased, it causes increase in adsorbed amount of sorbate on the surface of sorbent. At low pH value, metals have to compete with H^+^ ions for adsorption on sorbent surface since H^+^ ions are present in excess at that pH value. But when pH value is raised, it causes significant increase in adsorption due to attraction developed between negatively charged surfaces of sorbent by –OH groups and positively charged metal ions [23]. So, for cadmium, optimum pH range was found from 6 to 8; in this range, cadmium shows best adsorption behavior. When pH is further increased, there is decline in adsorption capacity due to formation of metal hydrides.

#### 3.3.3. Effect of Initial Concentration

Initial concentration of sorbate is another important parameter, which affects the adsorption phenomenon. For this purpose, initial concentration of cadmium was varied in range of 10–100 ppm by keeping all other parameters constant (Figure 3s Supplementary material).

Rapid increase in adsorption capacity was observed initially for adsorption of cadmium on *Saccharum arundinaceum* as vacant spaces were available on the surface of sorbent. So, rise in concentration also raised adsorption of sorbate on available sites [24]. Adsorbent readily occupies these adsorption sites, and adsorption capacity has positive influence of concentration in this range. Further increase in concentration from 60 to 100 ppm has no significant effect on adsorption phenomenon. Surface of adsorbent becomes saturated with sorbate, and after establishment of equilibria, increase in concentration has no significant influence on adsorption phenomenon. Previous studies also report that accommodation for sorbate decreases as concentration is very high due to unavailability of resident sites [25].

#### 3.3.4. Effect of Temperature

The effect of temperature on adsorption was studied by using temperature range 20, 30, 40, and 50°C with pH 6 (Figure 4s) (Supplementary material). Adsorption of cadmium onto *Saccharum arundinaceum* agrowaste was found to increase with increase in temperature. At high temperature, intraparticle diffusion increases and more adsorption sites are created which boost up adsorption phenomenon [26].

### 3.4. Equilibrium Isotherms

Adsorption system can be designed by adsorption isotherms commonly known as equilibrium isotherms which represent the amount of solute adsorbed per unit weight of sorbent [27]. These isotherms use equilibrium concentration of sorbent at constant temperature. In order to remove effluents from the system, particular design is optimized to generate proper correlation for experimental data which is called adsorption isotherm. Researches proposed many isotherms in this regard which are based on the adsorption system including Langmuir, Freundlich, Redlich–Peterson, Temkin, and Elovich [28, 29]. Sorption was carried out by employing linear as well as nonlinear adsorption models by varying initial concentration from 10 to 100 ppm.

#### 3.4.1. Freundlich Isotherm

Freundlich adsorption isotherm was developed for the heterogeneous system, and it gives concept of multilayer adsorption on the surface of sorbent (Figure 5s Supplementary material).

Parameters calculated for Freundlich isotherm by employing its linear and nonlinear form are given in Table 4. Freundlich isotherm was obtained by plotting log Cad versus log Ce. *K*_F_ and *n* are constants obtained from intercept and slope, respectively. Freundlich adsorption capacity (*K*_F_) is an indicator of a system, whether it is favorable for adsorption or not. Adsorption is considered promising if value of *K*_F_ is found in range of 1–20, and results reveal that in the present study, *K*_F_ was 9.5 and 12.2, respectively, for linear and nonlinear approaches of Freundlich adsorption isotherm. Similarly, adsorption intensity represented by *n* indicates fitness of model for adsorption purposes if value of *n* is above 1. Value of *R* ^2^ obtained from the plot is significant (0.9446) representing good fitness of this model for adsorption of cadmium onto *Saccharum arundinaceum*.

#### 3.4.2. Langmuir Isotherm

Langmuir adsorption isotherm is based on monolayer adsorption of metal ions on the surface of agrowastes, and energy of adsorption system is considered constant. In order to calculate Langmuir model, initial concentrations were changed from 10 to 100 ppm with 1 g sorbent amount and 1 h shaking time. Distribution of metal ions between liquid and solid surface was calculated by equations given in Table 1, employing linear and nonlinear forms of the model. Langmuir adsorption isotherm was obtained by plotting Ce/Cad versus Ce as shown in Figure 6s Supplementary material. *R* ^2^ value obtained for plot was found satisfactory showing fitness of model on the adsorption experiment. *Q*_o_ represents metal ion uptake per unit mass of adsorbent (mg/g) and b is Langmuir constant [15]. A dimensionless constant *R*_L_ is calculated by using Langmuir constant, and initial concentration represents model fitness for a particular system. If value of *R*_L_ falls between 0 and 1, the system is considered appropriate for adsorption purpose and Table 2 shows results which are in this range. Furthermore, experimental data and predicted results obtained for the present work were found in close correlation with low value of residual sum of square (0.006) making this model applicable for the present work.

#### 3.4.3. Dubinin–Radushkevich Isotherm

Dubinin–Radushkevich isotherm was designed as an empirical model for adsorption of vapors onto solid surface. It is successfully applied for adsorption of heterogeneous system including solid and liquid. This model is considered more general than Langmuir because in its derivation homogenous surface and constant sorption potential are not assumed [30]. The relationship given in Table 1s (supplementary data) was employed to relate ln Cad with *ε* ^2^, where *ε* ^2^ is Polanyi potential which is based on temperature, natural gas constant, and equilibrium concentration as given in the following equation:

(8) $$\begin{array}{c} \varepsilon =RT\text{⁡}\ln\left(1+\frac{1}{C_{e}}\right). \end{array}$$

Slope of the plot gives value of *k*_ad_ and the intercept is *q*_s_. The model showed good applicability on the adsorption system in nonlinear form with high value of *R* ^2^.

Dubinin–Radushkevich isotherm has found very promising applications for determination of nature of sorption, whether it is physical or chemical. For this purpose, *k*_ad_ obtained from the slope of the plot is used in the following equation:

(9) $$\begin{array}{c} E=\frac{1}{\sqrt{2k_{\mathrm{ad}}}}. \end{array}$$

The value of *E* calculated for the present research was 0.764 and suggests physical nature of sorption. Because the value of *E* below 8 kj/mol reflects physical sorption and 8–16 kj/mol reflects chemical sorption (Figure 7s Supplementary material).

#### 3.4.4. Temkin Isotherm

Temkin adsorption isotherm discusses interaction of sorbent and sorbate, and the model is based on assumption that heat of adsorption will not remain constant. It decreases due to interaction between sorbent and sorbate during adsorption phenomenon [31]. Linear and nonlinear forms of Temkin model are given in Table 1s (Supplementary data). Equilibrium constant of binding *K*_T_ provides information about binding energy, and *β* expresses heat of adsorption for a particular adsorption experiment (Figure 8s Supplementary material). Linear form of Temkin model is found more suitable with high value of binding constant as given in Table 2. The model indicates the exothermic nature of adsorption reaction as *B* > 0 which is an indicator of heat release during the process [32].

#### 3.4.5. Elovich Isotherm

According to Elovich model, mechanism of adsorption is based on chemical reactions which are responsible for adsorption. Plot of ln *C*_ad_/*C*_e_ versus *C*_ad_ gives *R* ^2^ value close to unity. *K*_E_ and *Q*_m_ are obtained from intercept and slope of plot, respectively. *K*_E_ shows initial sorption rate and *Q*_m_ is adsorption constant. Initial sorption rate obtained from linear form of Elovich model is quite high (35,100.411) as compared to nonlinear form (11.7891), so making linear form adequate to describe adsorption of cadmium onto *Saccharum arundinaceum*. Furthermore, *R* ^2^ value (0.9033) for linear form is also high than nonlinear form (0.835) (Figure 9s-Supplementary material).

### 3.5. Error Analysis for Equilibrium Isotherms

In order to check the fit of adsorption model to experimental data, error functions are used [33]. In the present work, six error functions were applied on linear and nonlinear form of data by minimizing the error function in a range of concentration used for analysis by employing solver add-in with Microsoft Excel 2010. Results for optimization of equilibrium isotherms by error analysis are given in Table 3. For meaningful results, a comparison of each error function for linear and nonlinear forms was made (Figure 10s Supplementary material).

For linear form of adsorption isotherms, a comparison of error functions reflects that Langmuir, Freundlich, and Elovich isotherms have good correlation with experimental values for the present adsorption study. These isotherms give low values for most of error functions. Applicability of these models for removal of cadmium ions from aqueous media is also studied by many researchers [34, 35].

 
*R* ^2^: Temkin > Langmuir > Elovich > Freundlich > Dubinin–Radushkevich
  RSS: Temkin > Dubinin–Radushkevich > Elovich > Freundlich > Langmuir
  ARE: Dubinin–Radushkevich > Temkin > Freundlich > Langmuir > Elovich
  EABS: Dubinin–Radushkevich > Langmuir > Temkin > Freundlich > Elovich
  Chi-square (*χ* ^2^): Elovich > Dubinin–Radushkevich > Freundlich > Temkin > Langmuir

Similar study was carried out by employing nonlinear form of adsorption models, and results are summarized below. Nonlinear form of Temkin isotherm was not found suitable for adsorption of cadmium onto *Saccharum arundinaceum* agrowaste because of high value of error functions. Freundlich and Elovich isotherms have been proved to be suitable models for this study with low value for error functions.

 
*R* ^2^: Langmuir > Freundlich > Dubinin–Radushkevich > Temkin > Elovich
  RSS: Temkin > Dubinin–Radushkevich > Freundlich > Langmuir > Elovich
  ARE: Temkin > Dubinin–Radushkevich > Langmuir > Elovich > Freundlich
  EABS: Temkin > Dubinin–Radushkevich > Elovich > Langmuir > Freundlich
  Chi-square (*χ* ^2^): Temkin > Freundlich > Dubinin–Radushkevich > Elovich > Langmuir

A comparison between linear and nonlinear approaches of each adsorption isotherm was also made to select the most appropriate form for adsorption study. Linear form of Freundlich adsorption isotherm was found superior over nonlinear form with small error functions in most of the cases.

 
*R* ^2^: Freundlich (linear approach) < Freundlich (nonlinear)
  RSS: Freundlich (linear approach) < Freundlich (nonlinear)
  ARE: Freundlich (linear approach) > Freundlich (nonlinear)
  EABS: Freundlich (linear approach) < Freundlich (nonlinear)
  Chi-square (*χ* ^2^): Freundlich (linear approach) < Freundlich (nonlinear)

For Langmuir adsorption isotherm, error function for nonlinear form was obtained high as compared with linear to exception of *R* ^2^.

 
*R* ^2^: Langmuir (linear approach) > Langmuir (nonlinear approach)
  RSS: Langmuir (linear approach) < Langmuir (nonlinear approach)
  ARE: Langmuir (linear approach) < Langmuir (nonlinear approach)
  EABS: Langmuir (linear approach) < Langmuir (nonlinear approach)
  Chi-square (*χ* ^2^): Langmuir (linear approach) < Langmuir (nonlinear approach)

Applicability of linear Dubinin–Radushkevich model was found better than nonlinear due to small value of error function (except ARE).

 
*R* ^2^: Dubinin–Radushkevich (linear approach) < Dubinin–Radushkevich (nonlinear)
  RSS: Dubinin–Radushkevich (linear approach) < Dubinin–Radushkevich (nonlinear)
  ARE: Dubinin–Radushkevich (linear approach) > Dubinin–Radushkevich (nonlinear)
  EABS: Dubinin–Radushkevich (linear approach) < Dubinin–Radushkevich (nonlinear)
  Chi-sq/*χ* ^2^: Dubinin–Radushkevich (linear approach) < Dubinin–Radushkevich (nonlinear)

Linear form of Elovich isotherm, which is also based on multilayer sorption on the surface of sorbent, shows small value of ARE and EABS, but other error functions were found lower for nonlinear form.

 
*R* ^2^: Elovich (linear approach) > Elovich (nonlinear approach)
  RSS: Elovich (linear approach) > Elovich (nonlinear approach)
  ARE: Elovich (linear approach) < Elovich (nonlinear approach)
  EABS: Elovich (linear approach) < Elovich (nonlinear approach)
  Chi-square (*χ* ^2^): Elovich (linear approach) > Elovich (nonlinear approach)

Linear approach for Temkin isotherm was found favorable for adsorption of cadmium ions onto *Saccharum arundinaceum* with low error function.

 
*R* ^2^: Temkin (linear approach) > Temkin (nonlinear approach)
  RSS: Temkin (linear approach) < Temkin (nonlinear approach)
  ARE: Temkin (linear approach) < Temkin (nonlinear approach)
  EABS: Temkin (linear approach) < Temkin (nonlinear approach)
  Chi-square (*χ* ^2^): Temkin (linear approach) < Temkin (nonlinear approach)

### 3.6. Thermodynamic Studies

Effect of temperature on adsorption was studied by using temperature range 20, 30, 40, and 50°C with pH 6 and variable initial concentration (30–120 ppm). Adsorption of cadmium onto *Saccharum arundinaceum* agrowaste was found to increase with increase in temperature. At high temperature, intraparticle diffusion increases and more adsorption sites are created which boost up adsorption phenomenon. Results for thermodynamic study are given in Table 4:

(10) $$\begin{array}{c} \text{Log}\frac{\text{Cad}}{\text{Ce}}-\frac{\Delta H}{2.303RT}+\frac{\Delta S}{2.303R}. \end{array}$$

Plot of log Cad/Ce versus 1/*T* was obtained with *R*-squared value 0.927. Slop and intercept provide value of ∆*H*° and ∆*S*°, respectively, as shown in (10).

∆*G*° was calculated by employing (1) given in Section 2 in temperature range 292–328 K. Results show a negative value for Gibb's free energy at all temperature ranges, and ∆*G*° increases with the increase in temperature. These negative values represent spontaneous nature as well as feasibility of adsorption reaction [36]. Decrease in ∆*G*° with the increase in temperature reflects better sorption at elevated temperature. The positive value for change in enthalpy is due to endothermic nature of adsorption of cadmium. Enthalpy was also found positive because randomness in system increases due to solid-liquid interaction during adsorption phenomenon. Sorption energy calculated by Dubinin–Radushkevich model was found below 1 which is an indication of physical nature of cadmium sorption on the surface of sorbent. *E* < 8 kJ/mol is representative of physical sorption, and *E* > 8–16 kJ/mol is due to chemical sorption [37]. For adsorption of cadmium value of *E* is found below 8, so adsorption of cadmium occurred on the surface and no chemical bonding took place between sorbent and sorbate. Similar results for adsorption of cadmium onto agrowaste were reported in the literature [38].

### 3.7. Adsorption Kinetics

Adsorption kinetics has prime importance in describing solute uptake rate and time required for adsorption process. In the present work, kinetic study was performed at different time intervals for cadmium adsorption by employing linear and nonlinear forms of pseudo-first- and second-order kinetics. Results indicate that amount of cadmium adsorbed increases with the increase in time interval; however, this increase was sharp in the start of reaction and gradually magnitude of adsorption decreases down. Initially, plenty of active sites were available on the surface of sorbent, so sharp rise in adsorption occurred, but these sites got occupied with the passage of time, so magnitude of adsorption gradually decreases [39].

#### 3.7.1. Pseudo-First-Order Kinetics

For pseudo-first-order kinetic model, log (*Q*_e_ − *Q*_t_) was plotted against time interval and value of *k* was obtained from slope of the line and *Q*_e_ from intercept. Initial sorption rate, *h*, was calculated by the following equation:

(11) $$\begin{array}{c} h=k_{2}Q_{e}^{2}. \end{array}$$

Poor correlation was obtained for linear form of the model with low value of *R* ^2^ (0.0918). Results indicate that adsorption of cadmium onto *Saccharum arundinaceum* does not follow pseudo-first-order kinetics. Nonlinear form of pseudo-first-order kinetics was obtained by using Microsoft Excel 2010 [40].

#### 3.7.2. Pseudo-Second-Order Kinetics

Second-order kinetics is applicable on small amount of initial concentration for determination of initial sorption rate. Different linear and nonlinear forms of pseudo-second-order kinetics are given in Table 3s (Supplementary material). These four linear forms of pseudo-second-order kinetic models were applied on experimental data, and Figures 11s–15s (Supplementary material) show results for these models. Coefficient of determination (*R* ^2^), found for type 1, was quite high indicating the best fitting of this model on adsorption data of cadmium. Results obtained for pseudo-second-order kinetic are given in Table 4s (Supplementary material). Theoretical results obtained for amount of cadmium adsorbed at equilibrium are found best fitted with experimental data for pseudo-second-order kinetics. For nonlinear form of pseudo-second-order, a computer-based procedure was used in Microsoft Excel 2010 using solver add-in method as reported in the literature [41]. For sorption of cadmium onto *Saccharum arundinaceum* pseudo-second-order kinetic may describe the method of adsorption in quite appropriate way as compared to pseudo-first-order approach. Furthermore, nonlinear form of pseudo-second-order gives close results to experimental data.

### 3.8. Effect of Interfering Ions

Process of adsorption becomes complicated in case of multicomponent adsorption as many interactions of sorbent and sorbate are involved. Effect of interfering ions was measured by observing adsorption of one metal ion, and then, by addition of interfering ion change in adsorption capacity was noted. Interfering effect was calculated by employing the following equation:

(12) $$\begin{array}{c} \text{interfering}\;\;\mathrm{capacity}=\frac{C_{\text{mix}}}{C}, \end{array}$$

where *C*_mix_ is the % adsorption of mixture of two metal ions and *C* is the % adsorption of pure metal on selected sorbent. If the value of *C*_mix_/*C* is found equal to 1, then there is no effect of interfering ions on adsorption phenomenon. However, if it is found less than 1, then adsorption capacity is found to be affected by addition of these interfering ions. To study interference, ions were divided into three categories as monovalent, bivalent, and trivalent ions based on valences of ions. One ion was selected from each class of ions to check the interfering effect on adsorption of cadmium ions. Metals are attached on the surface of sorbent through electrostatic forces, and competition of metals for sorbent place is mainly based on metal ion charge and its attraction towards functional groups present on the surface on adsorbent. Results for interference of metal ions are summarized in Table 5. Metals with high charge value were found to have maximum effect on adsorption of cadmium as compared with those with low charge. Anions have also found to affect the adsorption phenomenon of metal ions but their interference is comparatively very less than cations. Adsorption of anions on sorbent is dependent on charge present on the surface of sorbent. Since negatively charged hydroxyl groups are present on the surface of adsorbent, adsorption of anions is not as favored as cations [42].

## 4. Conclusion

Removal of cadmium was performed by employing the stem powder of *Saccharum arundinaceum*. In order to generate proper correlation for the removal of cadmium, five adsorption isotherms were applied on experimental data including Freundlich, Langmuir, Dubinin–Radushkevich, Elovich, and Temkin. Error analysis provides information about fitness of these models on experimental data. The model with minimum error was selected best for adsorption data. Order of equilibrium isotherms according to increasing RSS value was found as Temkin > Dubinin–Radushkevich > Elovich > Freundlich > Langmuir.

Linear form of Freundlich and Langmuir models was found best fitted with minimum value of error. Effect of temperature on cadmium adsorption was investigated by thermodynamic analysis, and it was found to increase with increase in temperature. Gibb's free energy (∆*G*° = −612.34 at 303 K) revealed spontaneous nature of sorbent-sorbate binding reaction, and it followed pseudo-second-order kinetics.

## Figures and Tables

**Table 1:** Identified functional groups present in *Saccharum arundinaceum* by FTIR spectroscopy.

Possible functional groups	Raw (cm^−1^)	Base treated (cm^−1^)	Acid treated (cm^−1^)
–OH stretching	3309–3751	3211–3400	
=CH			3294
C–H	2858–2918 (bifurcate)	2910	2922
C=O			
Secondary amide	1645	1608	1654.92
–NH			
C–O	1107	1222	
C–N	1051	1056	1043

**Table 2:** Linear and nonlinear parameters of isothermal models for sorption of Cd onto *Saccharum arundinaceum*

Models	Linear method	Nonlinear method
*Freundlich*		
*K*_F_ (mg/g) (L/mg)^n^	9.4558	12.2001
*N*	2.42	3.0271
*R* ^2^	0.9446	

*Langmuir*		
*Q*_o_ (mg/g)	48.309	48.0821
*b* (L/mg)	0.1446	0.1461
*R*_L_	0.408	0.406
*R* ^2^	0.9958	

*Dubinin–Radushkevich*		
*k*_ad_(mol^2^/kJ^2^)	0.6543	3.1816
*q*_s_ (mg/g)	34.3533	39.667
*R* ^2^	0.7912	

*Temkin*		
*Β* (J/mol)	264.39	161.8954
*K*_T_	1.0227	0.38
*R* ^2^	0.9852	

*Elovich*		
*Q*_m_ (mg/g)	16.58	17.2006
*K*_E_ (L/mg)	1.1677	0.6631
*R* ^2^	0.9433	

**Table 3:** Error functions for optimization of equilibrium isotherms.

Error functions	*R* ^2^	ERRSQ/RSS	ARE	EABS	Chi-square (*χ* ^2^)
*Linear approach*					
Freundlich	0.8092	0.0261	−0.8917	−1.9864	3.7154
Langmuir	0.9958	0.0060	−1.2623	−0.0054	0.0106
Dubinin–Radushkevich	0.0523	17.6722	26.6963	9.2320	4.9631
Elovich	0.9432	1.8130	−196.878	−3.9160	10.0831
Temkin	1	18.9131	−0.0723	−0.0055	0.6727

*Nonlinear approach*					
Freundlich	0.9476	69.1613	−4.9554	−1.4878	40.3303
Langmuir	0.9893	14.0441	1.0363	0.6722	0.5029
Dubinin–Radushkevich	0.9148	166.7881	10.4851	9.4789	11.7308
Elovich	0.8354	1.0911	0.7977	5.3805	0.5322
Temkin	0.8359	766.4798	38.3613	49.2605	65.3952

**Table 4:** Thermodynamic parameters for adsorption of cadmium onto *Saccharum arundinaceum*.

Parameters	Temperature (K)	Results
	293	−591.24
∆*G*° (kJ/mol)	303	−612.34
	313	−633.44
	328	−665.09
∆*H*° (kJ/mol)		26.99
∆*S*° (kJ/mol·K)		2.11
Sorption energy (kJ/mol)		0.764

**Table 5:** Effect of interfering cations and anions on % adsorption of cadmium.

Interfering cations		Effect on adsorption of Cd^+2^
Monovalent	K^+1^	0.958
Bivalent	Ca^+2^	0.644
Trivalent	Cr^+3^	0.491

Interfering anions	Cl^−1^	0.945
	NO_3_^−1^	0.881
	SO_4_^−2^	0.907

## Data Availability

The data used to support the findings of this study are available from the corresponding author upon request.

## Supplementary Materials

Supplementary Materials Figures 1s–4s: effect of different parameters (contact time, pH, initial concentration of cadmium, and temperature) which were adjusted during adsorption for maximum removal of cadmium from aqueous media. Figures 5s–9s: graphical results of isothermal study. Data derived from these figures are present in Table 4 of the main manuscript. Figure 10s: thermodynamic studies. Results of Figures 11s–15s of kinetic studies are described in these figures. Linear and nonlinear forms of equilibrium isotherms are given in Tables 1s and 2s. Table 2s provides information about equations of error functions applied on the results. Tables 3s and 4s describe kinetic studies and formulas applied on work for calculation of pseudo-first- and second-order kinetics.
