# Supplementary material for: Study of Isothermal, Kinetic, and Thermodynamic Parameters for Adsorption of Cadmium: An Overview of Linear and Nonlinear Approach and Error Analysis
Source: Bioinorg Chem Appl. 2018 Jul 3;2018:3463724. doi: 10.1155/2018/3463724 (PMC6051333; doi:10.1155/2018/3463724)
Supplement: Supplementary Materials — Figures 1s–4s: effect of different parameters (contact time, pH, initial concentration of cadmium, and temperature) which were adjusted during adsorption for maximum removal of cadmium from aqueous media. Figures 5s–9s: graphical results of isothermal study. Data derived from these figures are present in Table 4 of the main manuscript. Figure 10s: thermodynamic studies. Results of Figures 11s–15s of kinetic studies are described in these figures. Linear and nonlinear forms of equilibrium isotherms are given in Tables 1s and 2s. Table 2s provides information about equations of error functions applied on the results. Tables 3s and 4s describe kinetic studies and formulas applied on work for calculation of pseudo-first- and second-order kinetics. [file 3463724.f1.docx]

**SUPPLEMENTARY MATERIALS**

**Study of Isothermal, Kinetic and Thermodynamic Parameters for Adsorption of Cadmium: An overview of Linear and Non-Linear Approach and Error Analysis**

Fozia Batool^1*^, Jamshed Akbar^1^, Shahid Iqbal^1^, Sobia Noreen^1^ , Syed Nasir Abbas Bukhari^2*^

***^1^*** *Department of Chemistry, University of Sargodha, Sargodha 40100 Pakistan*

*^2^ Department of Pharmaceutical Chemistry, College of Pharmacy, Jouf University, Aljouf, Sakaka 2014, Saudi Arabia*

**CONTENTS IN SUPPLEMENTARY DATA**

| Supplementary Material | Description |
| --- | --- |
| Fig 1s-4s | These figures describe effect of different parameters (Contact time, pH, initial concentration of cadmium and temperature) which were adjusted during adsorption for maximum removal of cadmium from aqueous media. |
| Fig 5s-9s | These figures describe graphical results of isothermal study. Data derived from these figures in present in table 4 of main manuscript. |
| Fig 10s | It describes thermodynamic studies. |
| Fig 11s-15s | Results of kinetic studies are described in these figures. |
| Table 1s and 2s | Linear and non-linear forms of equilibrium isotherms are given in table. Table 2s provide information about equations of error functions applied on the results. |
| Table 3s and 4s | These tables describe kinetic studies and formulas applied on work for calculation of pseudo-first and second order kinetics. |

**Fig 1s (Supplementary material)**

**Fig 1s:Effect of Contact Time (10-100 mint) on Adsorption Potential of *Saccharumarundinaceum***

**Fig 2s (Supplementary material)**

**Fig 2s: Effect of pH(2-10) on Adsorption Potential of *Saccharumarundinaceum***

**Fig 3s (Supplementary material)**

**Fig 3s: Effect of Initial Concentration (10-100ppm) on Adsorption Potential of *Saccharumarundinaceum***

**Fig 4s (Supplementary material)**

**Fig 4s:Effect of Temperature (20-50 ºC) on Adsorption Potential of *Saccharumarundinaceum***

**Fig 5s (Supplementary material)**

**Fig 5s: Freundlichadsorption isotherm of Cadmium on 1 g of SA at 60 minutes shaking time and 10-100 ppm initial concentration**.

**Fig 6s (Supplementary material)**

**Fig 6s: Langmuir adsorption isotherm of Cadmium on 1g of SA at 60 minutes shaking time and 10-100ppm initial concentration.**

**Fig 7s (Supplementary material)**

**Fig 7s: Dubinin–Radushkevichadsorptionisotherm of Cadmium on 1 g of SA at 60 minutes shaking time, 25ºC temperature and 10-100 ppm initial concentration.**

**Fig 8s (Supplementary material)**

**Fig 8s: Temkinadsorption isotherm of Cadmium on 1 g of SA at 60 minutes shaking time and 10-100ppm initial concentration.**

**Fig 9s (Supplementary material)**

**Fig 9s: Elovichadsorption isotherm of Cadmium on 1 g of SA at 60minutes shaking time, 25ºC temperature and 10-100 ppm initial concentration.**

**Fig 10s (Supplementary material)**

**Fig 10s: Plot of 1/T vs log Cad/Ce for Thermodynamic studies at pH 6 and temperature range 20-50** ºC

**Fig 11s (Supplementary material)**

**Fig 11s Plot of log (Qe-Qt) vs. time for Pseudo-first order kinetic model (Linear Form)**

**Fig 12s (Supplementary material)**

**Fig 12s: Plot of t/Q_t_ vs. time for Pseudo-second order kinetic model (Type 1)**

**Fig 13s (Supplementary material)**

**Fig 13s:Plot of 1/Q_t_ vs. 1/t for Pseudo-second order kinetic model (Type 2)**

**Fig 14s (Supplementary material)**

**Fig 14s: Plot of Q_t_/t vs. Q_t_ for Pseudo-second order kinetic model (Type 3)**

**Fig 15s (Supplementary material)**

**Fig 15s: Plot of Q_t_ vs. Q_t_/t for Pseudo-second order kinetic model (Type 4)**

**Table 1s:** Equilibrium Isotherms with their Linear & Non-Linear Forms applied on Work

| **Equilibrium Isotherms** | **Linear Form** | **Non-Linear Form** |
| --- | --- | --- |
| **Freundlich** | $\text{LogCad = LogK}_{\text{F}}\text{+}\frac{1}{\text{n}}\text{logC}_{\text{e}}$ | ^^ |
| **Langmuir** | __ | $\text{Cad = }\frac{\text{Q}_{_{\text{o}}}\text{bC}_{\text{e}}}{\text{1+bC}_{\text{e}}}$ |
| **Dubinin-Radushkevich** | $\text{ln(Cad) = ln(q}_{\text{s}}\text{)-k}_{\text{ad}}\text{ε}^{2}$ | $\text{Cad = }\left(\text{q}_{\text{s}}\right)\text{exp}\left(\text{-k}_{\text{ad}}\text{ε}^{2}\right)$ |
| **Temkin** | $\text{Cad = }\frac{\text{RT}}{\Delta\text{Q}}\text{ lnK}_{\text{T}}\text{+ (}\frac{\text{RT}}{\Delta\text{Q}}\text{) lnC}_{\text{e}}$ | $\text{Cad = }\frac{\text{RT}}{\Delta\text{Q}}\text{ lnK}_{\text{T}}\text{C}_{\text{e}}$ |
| **Elovich** | **$\text{ln}\frac{\text{Cad}}{\text{Ce}}\text{ = lnK}_{\text{E}}\text{Q}_{\text{m}}\text{- }\frac{\text{Cad}}{\text{Q}_{\text{m}}}$** | $\frac{\text{Cad}}{\text{q}_{\text{m}}}\text{ = K}_{\text{E}}\text{C}_{\text{e}}\text{exp (- }\frac{\text{q}_{\text{e}}}{\text{q}_{\text{m}}}\text{)}$ |

**Table 2s:**Error Functions and their Equations

| **Error Functions** | **Equation Applied for Calculation** |
| --- | --- |
| **The Sum Square Error (SSE)** |  |
| **The Average Relative Error (ARE)** | $\text{ARE = }\frac{100}{\text{n}}\sum_{\text{i=1}}^{\text{n}} \left\vert\frac{\text{q}_{\text{e.meas-}}\text{q}_{\text{e.calc}}}{\text{q}_{\text{e.meas}}}\right\vert_{\text{i}}$ |
| **The Sum of Absolute Error (EABS)** | $\text{EABS = }\sum_{\text{i=1}}^{\text{n}} \left\vert\text{q}_{\text{e.meas}}\text{- q}_{\text{e.calc}}\right\vert$ |
| **Coefficient of Determination (R^2^)** | $\text{R}^{2}\text{ = }\frac{\left(\text{q}_{\text{e.meas}}\text{- }\overline{\text{q}_{\text{e.calc}}}\right)^{2}}{\sum\left(\text{q}_{\text{e.meas}}\text{- }\overline{\text{q}_{\text{e.calc}}}\right)^{2}\text{+}\left(\text{q}_{\text{e.maes}}\text{- q}_{\text{e.calc}}\right)^{2}}$ |
| **Non-linear Chi-Square Test (Chi-Sq/χ^2^)** | $\begin{matrix} \\ \text{Chi-Sq = }\sum_{\text{i=1}}^{\text{n}} \frac{\left(\text{q}_{\text{e.calc}}\text{- q}_{\text{e.meas}}\right)^{2}}{\text{q}_{\text{e.meas}}} \end{matrix}$ |
| **Average Percentage Error (APE)** | $\begin{matrix} \\ \text{APE(\%) = }\frac{\sum_{\text{i=1}}^{\text{n}} \left\vert\frac{\text{(q}_{\text{e.meas}}\text{- q}_{\text{e.calc}}\text{)}}{\text{q}_{\text{e.meas}}}\right\vert}{\text{N}}\text{ X 100} \end{matrix}$ |

**Table 3s:** Pseudo-First-Order and Second-Order Kinetic Models Applied on Present Work

| **Pseudo-Second-Order Model Form** | | **Equation** | **Plot** | **Parameters** |
| --- | --- | --- | --- | --- |
| **Linear Forms** | Type 1 | $\frac{\text{t}}{\text{Q}_{\text{t}}}\text{= }\frac{1}{\text{k}_{2}\text{Q}_{\text{e}}^{2}}\text{ + (}\frac{1}{\text{Q}_{\text{e}}}\text{) t}$ | t/Q_t_ vs. t | Q_e_ = 1/Slope  h = 1/Intercept  k = (slope)^2^/Intercept |
|  | Type2 | $\frac{1}{\text{Q}_{\text{t}}}\text{ = (}\frac{1}{\text{k}_{2}\text{Q}_{\text{e}}^{2}}\text{)}\frac{1}{\text{t}}\text{ + (}\frac{1}{\text{Q}_{\text{e}}}\text{) }$ | 1/Q_t_ vs. 1/t | Q_e_ = 1/Intercept  h = 1/Slope  k = (Intercept)^2^/Slop |
|  | Type 3 | $\frac{\text{Q}_{\text{t}}}{\text{t}}\text{ = kQ}_{e}^{2}\text{ - kQ}_{\text{e}}\text{Q}_{\text{t}}$ | Q_t_/t vs. Q_t_ | Q_e_ = -Intercept/Slop  h = Intercept  k = (Slop)^2^/Intercept |
|  | Type 4 | $\text{Q}_{\text{t}}\text{ = Q}_{\text{e}}\text{- (}\frac{1}{\text{kQ}_{\text{e}}}\text{)}\frac{\text{Q}_{\text{t}}}{\text{t}}$ | Q_t_ vs. Q_t_/t | Q_e_ = Intercept  h = -Intercept/Slop  k = -1/(Intercept x Slop) |
| **Pseudo-Second-Order Non-Linear Form** | | $\text{Q}_{\text{t}}\text{=}\frac{\text{k}_{2}\text{Q}_{\text{e}}^{2}\text{t}}{\text{1+ k}_{2}\text{Q}_{\text{e}}\text{t}}$ | 1/Q_t_ vs. 1/t | Q_e_ = 1/Intercept  h = 1/Slope  k = (Intercept)^2^/Slop |
| **Pseudo-First-Order Lineal Form** | | $\text{ln(Q}_{\text{e}}\text{-Q}_{\text{t}}\text{) = ln(Q}_{\text{e}}\text{)-k}_{1}\text{t}$ | ln(Q_e_-Q_t_)vs. t | k = Slop |
| **Pseudo-First-Order Non-Lineal Form** | | $\text{Q}_{\text{t}}\text{= Q}_{\text{e}}\text{(1-e}^{\text{-k}_{1}\text{t}}\text{)}$ | ln(Q_e_-Q_t_)vs. t | k= Slop |

**Table 4s:** Pseudo-First-Order and Second-Order Kinetic Parameters obtained by using linear and Non-Linear methods

| **Pseudo-second order kinetics** | | Parameters | | |
| --- | --- | --- | --- | --- |
|  |  | Qe (mg/g) | h (mg/g.min) | k (g/mg.min) |
| **Linear Form** | Type 1 | 20.36 | 4.14 | 0.0099 |
|  | Type 2 | 19.37 | 6.42 | 0.017 |
|  | Type 3 | 19.84 | 5.3016 | 0.0135 |
|  | Type 4 | 19.44 | 6.266 | 0.0165 |
| **Non-Linear Form** | | 19.6142 | 5.79 | 0.0150 |
| **Pseudo-first order kinetics** | | | | |
| **Linear Form** | |  | 7.63 | 0.0248 |
| **Non-Linear Form** | | 18.1707 | 53.54 | 0.1621 |
